# Experimental data on Helically Coiled Oscillating Heat Pipe (HCOHP) design and thermal performance

**DOI:** 10.1016/j.dib.2020.106505

**Published:** 2020-11-05

**Authors:** Siegfried K. Yeboah, Jo Darkwa

**Affiliations:** a Department of Architecture and Built Environment, Faculty of Science and Engineering, The University of Nottingham Ningbo China,199 Taikang East Road, Ningbo 315100, PR China; b Department of Architecture and Built Environment, the University of Nottingham, University Park, Nottingham, NG7 2RD, UK

**Keywords:** Design Parameters, Experimental Heat Transfer Data, Oscillating Heat Pipe, Thermal Management, Thermal Resistance, Working Fluids

## Abstract

Experimental and derived data from three Helically Coiled Oscillating Heat Pipes (HCOHPs) charged with ethanol, methanol and deionized water working fluids respectively are presented. The data was obtained from prototypes of the HCOHPs fabricated out of copper and tested under laboratory conditions. The primary data presented covers the HCOHP aspects, charging of the working fluid and temperature measurements from Omega K-type Thermocouples installed on the evaporators, condensers, adiabatic sections, and on the cylindrical copper vessel integrated with it. The derived data covers the HCOHPs performances and thermal contact resistance experienced during laboratory testing. The data on the aspects and charging of the working fluid provides useful information for the validation of design parameters of other heat pipes. The measured temperature data and the derived performance data can used to validate the performance of heat pipes in other studies and to depict performance profiles in standard text and reference books. The nature of the data presented as a whole would be useful for comparative analysis involving heat pipes and other passive heat transfer devices.

## Specifications Table

**Table:** 

Subject	Mechanical Engineering
Specific subject area	Thermal and Fluids Engineering
Type of data	Table Image Chart Graph Figure
How data were acquired	Primary data obtained from Omega K type thermocouples, Yokogawa MV 2000 data logger, Personal Computer, Vacuum Pump for evacuating HCOHPs, HCOHPs pressure testing using DynAir compressor, and derived data from primary data applied to established equations.
Data format	Raw Analyzed
Parameters for data collection	Data was collected under ambient laboratory conditions. Standard atmospheric pressure was approximately 101,325Pa. Ambient laboratory temperature ranged between 18–20 °C.
Description of data collection	The primary raw temperature data was obtained by connecting three Omega K type thermocouples to the evaporator and condenser coils, respectively. Two Omega K type thermocouples were connected to the two adiabatic sections of each HCOHP and two more were connected to the inside and two to the outside of the cylindrical copper vessel for the heat transfer to the evaporator coils. The Omega K type thermocouples were connected to the Yokogawa MV 2000 data logger which was connected to a personal computer to log the raw temperature data every 5.00 s. The HCOHP pressure testing data was obtained directly from the pressure gauge on the DynAir compressor. The evacuation pressure data was obtained directly from the Vacuum Pump. The raw primary temperature data along with the property and aspect data of the set-up were applied to equations for the derivation of the thermal resistance, thermal contact resistance and heat input power.
Data source location	Institution: The University of Nottingham Ningbo China City/Town/Region: Ningbo, Zhejiang Province. Country: P.R. China
Data accessibility	Repository name: Mendeley Data Data identification number: doi: 10.17632/wnf5jwzp3c.2 Direct URL to data: http://dx.doi.org/10.17632/wnf5jwzp3c.2
Related research article	S. K. Yeboah, J. Darkwa. Thermal performance of a novel helically coiled oscillating heat pipe (HCOHP) for isothermal adsorption. An experimental study. International Journal of Thermal Sciences, Volume 128, June 2018, Pages 49-58. https://doi.org/10.1016/j.ijthermalsci.2018.02.014

## Value of the Data

• The raw primary data shows the temperature profiles of the evaporator, condenser, and adiabatic sections of the Helically Coiled Oscillating Heat Pipes along with the temperature profiles of the inner and outer surfaces of the vessel transferring heat to the evaporators. These raw primary data can be useful in comparative analysis involving heat pipes using similar working fluids or even having different configurations. The derived primary data representing results associated with the thermal performance and thermal contact resistance can be suitably used as data for thermal performance comparison and verification. Also, the trends from the data can be used to show typical performance profiles in standard text and reference books for students and practitioners.
• Researchers, experts and industry practitioners in fluids and thermal engineering can benefit from such data for comparative analysis. They can also use this data to validate the performance of their heat exchangers and other passive heat transfer devices.
• This data can be used to visualize thermal performance of the HCOHPs as obtained from the experiment for illustrations in standard text and reference books. Data on the design parameters can be used in the calibration of other heat pipes so they have representative parameters.
• The data supports the design of a passive heat transfer device for temperature flattening in adsorption processes. Comparison with other systems geared towards similar aim will be invaluable to other researchers to gauge how much contribution or improvement they can gain using their own design.

## 1 Data Description

The data presented here are for ethanol, methanol and deionized water charged Helically Coiled Oscillating Heat Pipes (HCOHPs) tested under laboratory conditions at three different heat inputs [1]. The tests were designated Run 1 or (R1), Run 2 or (R2) and Run 3 or (R3) representing maximum heat input element temperatures of 100 °C, 125 °C and 192 °C, respectively. The ethanol, methanol, and deionized water HCOHPs were referred to as EOHP, MOHP and WOHP, respectively. The raw primary data are in the Mendeley repository as Excel Files [2] captioned HCOHP Primary Data – Run 1, HCOHP Primary Data – Run 2, and HCOHP Primary Data – Run 3. Within each Excel File are the raw temperature data in degrees Celsius (°C) from the three Omega K type thermocouples connected to the evaporator and condenser respectively and numbered 1 to 3 accordingly. Also, here are the raw temperature data for the adiabatic sections and the temperature data obtained from the inner and outer surface of the cylindrical copper vessel that transferred heat to the evaporators. The first tab of each HCOHP Primary Data Excel file has all the temperature data obtained for the EOHP, MOHP, WOHP, the inner and outer vessel temperatures. Subsequent tabs are captioned with the HCOHP type and the test run number for those specific raw primary data. Above the data are the Yokogawa MV2000 channel numbers for data collection. The data was obtained at sampling time interval of 5.00 s.

**Fig. 1:**
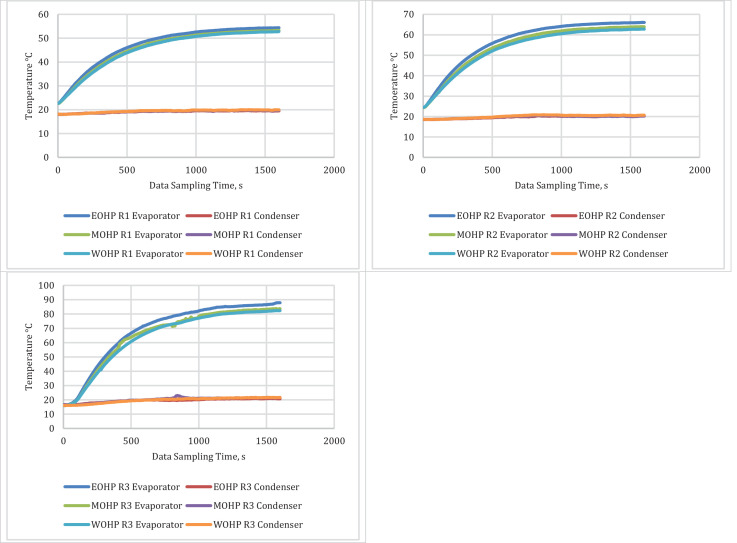
Average Evaporator and Condenser Temperatures in °C for the three test runs.

The derived results from the primary data are in the Mendeley repository as Excel files [2] labelled HCOHP Derived Data – Run 1, HCOHP Derived Data – Run 2, and HCOHP Derived Data – Run 3. Here, each file has one spreadsheet tab with data generated from the average evaporator and condenser temperatures of the EOHP, MOHP and WOHP tested under the three conditions in the laboratory. The inner and outer vessel temperatures obtained were also averaged to determine the average heat output from the cylindrical copper vessel. The temperature measurements obtained from the three Omega K type thermocouples connected to the evaporators and condensers of the EOHP, MOHP and WOHP were averaged for each test run and presented in Fig. 1.

**Fig. 2:**
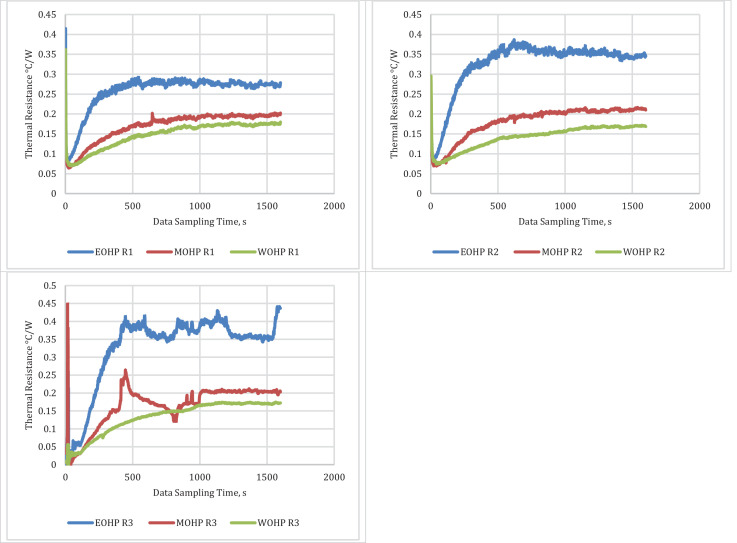
Transient Thermal Resistance Profiles for the HCOHPs during the three test runs.

The average temperature differences between each evaporator and condenser of the HCOHPs were used to determine the transient overall thermal resistances shown in Fig. 2. The overall thermal resistance data obtained makes it possible to compare the performance of the HCOHPs against each other. In the derived dataset, the various temperature differences, the thermal contact resistances, and the heat fluxes from the copper vessel are presented.

## 2 Experimental Design, Materials and Methods

### 2.1 Fabrication data

**Fig. 3:**
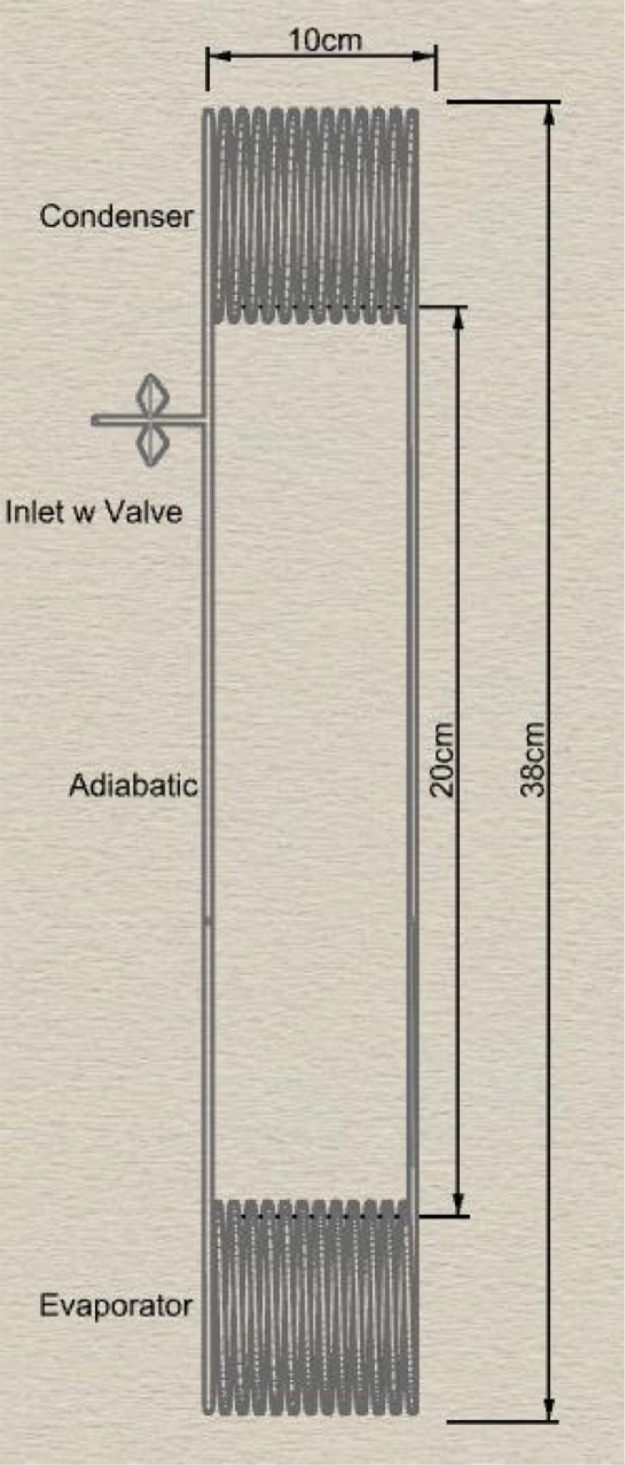
Sketch of the HCOHP with annotation and dimensions.

Fig. 3 shows the sketch of the helically coiled closed loop oscillating heat pipe (HCOHP) with relevant dimensions. The HCOHPs were fabricated out of copper pipe with the specifications in Table 1.

**Table 1:** Specifications for the HCOHPs.

Parameter	Value	Units
Inner Diameter	2	mm
Thickness	1	mm
Diameter of Coil	8	cm
Length of Compressed Coil	10	cm
Number of Turns	10	-

### 2.2 Pressure testing

**Fig. 4:**
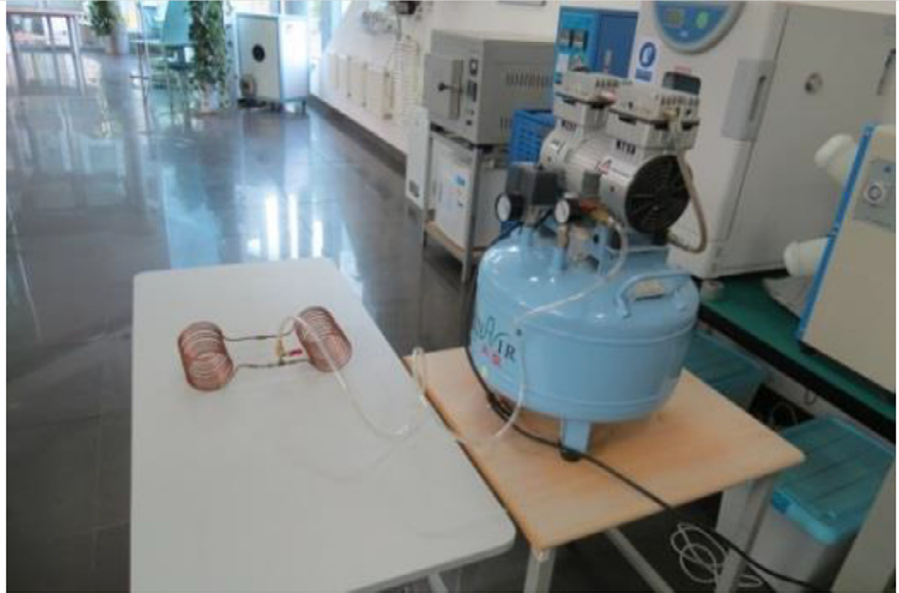
Pressure Testing of the HCOHPs.

Prior to experimentally investigating their thermal performances, they were pressure tested using a DynAir compressor (See Fig. 4). Here, a tube was connected to the compressor and the inlet valve of the HCOHP. The compressor was then turned on to generate a maximum air pressure of about 8 kPa. The compressor valve was then opened to allow the air to fill each HCOHP. A maintained pressure of about 3bar (∼41 psi) was recorded for each HCOHP tested. Pressure testing was essential to ensure that vacuum can be created within the pipe. Khandekar et al. [3] for instance fitted a T-connector on their PHP with a filling/metering valve, tested the final assembly under vacuum, and found that a pressure of 10^−4^ mbar could be easily maintained.

### 2.3 Charging the HCOHPs with Working Fluids

**Fig. 5:**
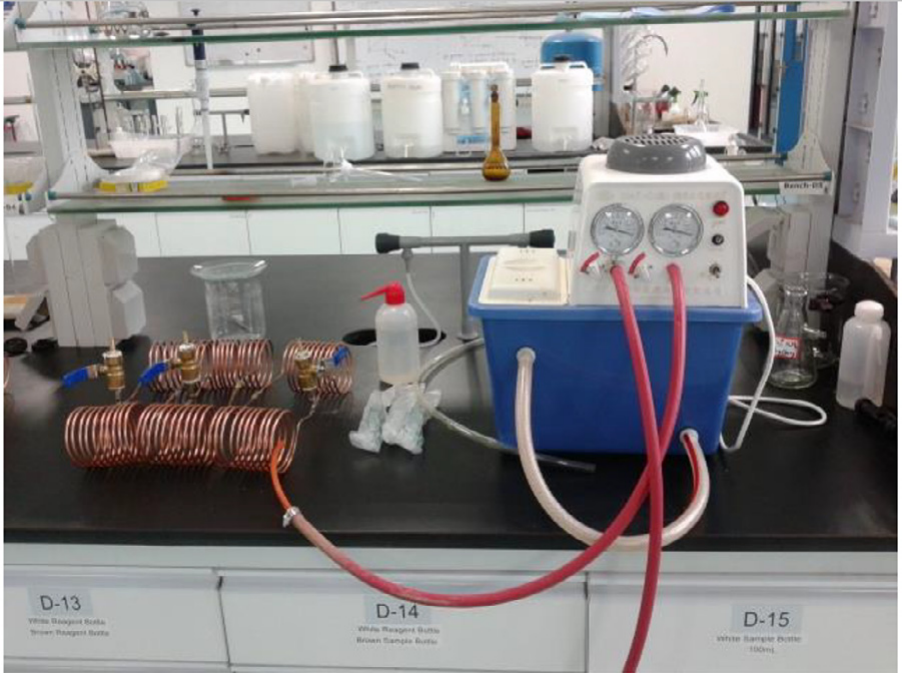
Evacuation and Filling of HCOHPs in the Laboratory.

Before charging the HCOHPs with working fluid, they were evacuated by a maximum pressure of about ∼0.001325 MPa using a vacuum pump (See Fig. 5), under standard atmospheric pressure of approximately 101,325 Pa.

The HCOHPs were then weighed empty and their individual weights recorded as shown in Table 2. Once evacuated, the HCOHPs were then fully filled with working fluid using a laboratory syringe and the volume that completely filled them was recorded. The fully filled HCOHPs were then weighed again. They were then evacuated completely using the vacuum pump before filling to about 60% volume with working fluid using a laboratory syringe in a fume cupboard (under ambient laboratory temperature and atmospheric pressure). Once again, the respective weights of the partially filled HCOHPs were recorded. According to Senjaya and Inoue [4] high heat transfer rate occurs when OHPs are charged at the optimum filling ratios (about 50–60%), which are higher than those of conventional heat pipes. The filling process involved evacuating the HCOHP and then closing the inlet valve to maintain the evacuation pressure. The required working fluid volume was subsequently drawn from a container using the laboratory syringe. The laboratory syringe containing the working fluid was then securely connected to the inlet valve of the HCOHP and the valve subsequently opened to draw the working fluid into the HCOHP. Once all the working fluid was automatically drawn into the HCOHP (due to the vacuum pressure), the inlet valve was closed to ensure the HCOHP maintained the vacuum pressure. Leaks were checked by immersing the whole HCOHP in a water bath at ambient temperature.

**Table 2:** HCOHP Working Fluid Charging Data.

Heat Pipe	Working Fluid	Dry HCOHP Weight, kg	Weight @ 60% Filled Volume, kg	Mass of Working Fluid in HCOHP, kg	Fully Filled Volume, ml	Partially Filled (60%) Volume, ml	Evacuation Pressure, MPa
EOHP 1	Ethanol	0.679	0.696	0.017	∼26	∼16	∼0.001325
EOHP 2	Ethanol	0.671	0.683	0.012	∼26	∼16	∼0.001325
EOHP 3	Ethanol	0.681	0.693	0.012	∼26	∼16	∼0.001325
MOHP 1	Methanol	0.679	0.691	0.012	∼26	∼16	∼0.001325
MOHP 2	Methanol	0.677	0.689	0.012	∼26	∼16	∼0.001325
MOHP 3	Methanol	0.677	0.691	0.014	∼26	∼16	∼0.001325
WOHP 1	Deionized Water	0.637	0.656	0.019	∼26	∼16	∼0.001325
WOHP 2	Deionized Water	0.637	0.648	0.011	∼21	∼12.6	∼0.001325
WOHP 3	Deionized Water	0.636	0.657	0.021	∼25	∼15	∼0.001325

The thermophysical properties of the working fluids along with their Figures of Merit are presented in Table 3.

**Table 3:** Thermophysical Properties and Figure of Merit for the Selected Working Fluids at 30 °C.

Working Fluid	Temperature, °C	Density (kg/m^3^), $\;\rho_{l}$	Latent Heat of Evaporation(kJ/kg), λ	Surface Tension (N/m), σ	Liquid Viscosity (cP), $\mu_{l}$	Figure of Merit M (W/m^2^)	Comments
Ethanol	30	781	888.60	0.024	1.02	1.6 × 10^7^	Figure of merit calculated with data from Reay et al. [5]
Methanol	30	782	1155.00	0.022	0.52	3.78 × 10^7^	
Water	30	996	2430.50	0.071	0.80	2.15 × 10^8^	

### 2.4 Data collection and measurement devices

**Fig. 6:**
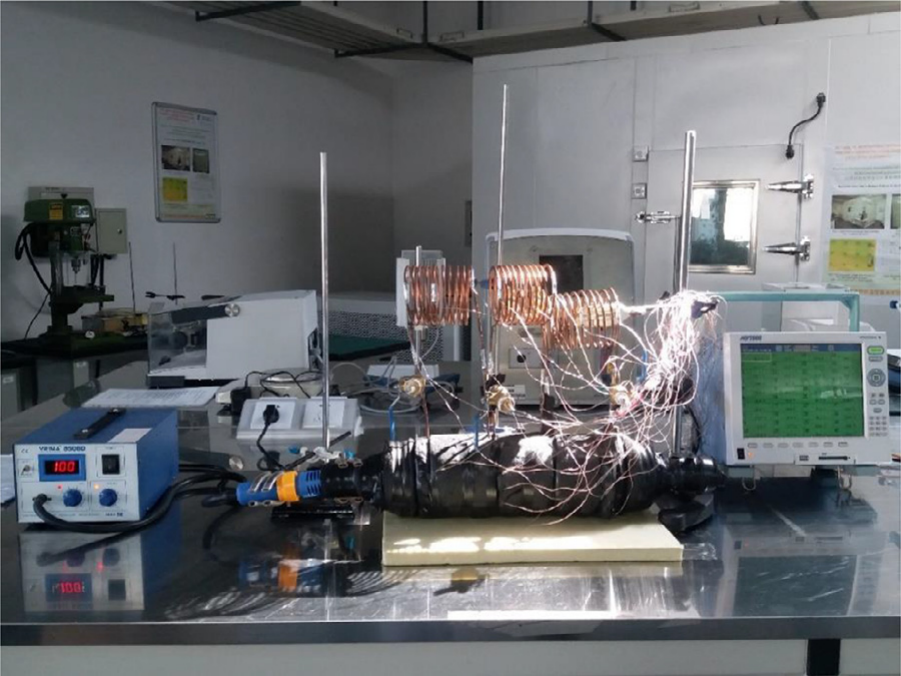
HCOHP Test Rig in the Laboratory.

The primary data collection devices used in this study was Omega K-type thermocouples, a Yokogawa MV2000 and a desktop computer as shown in the test rig in Fig. 6.

**Fig. 7:**
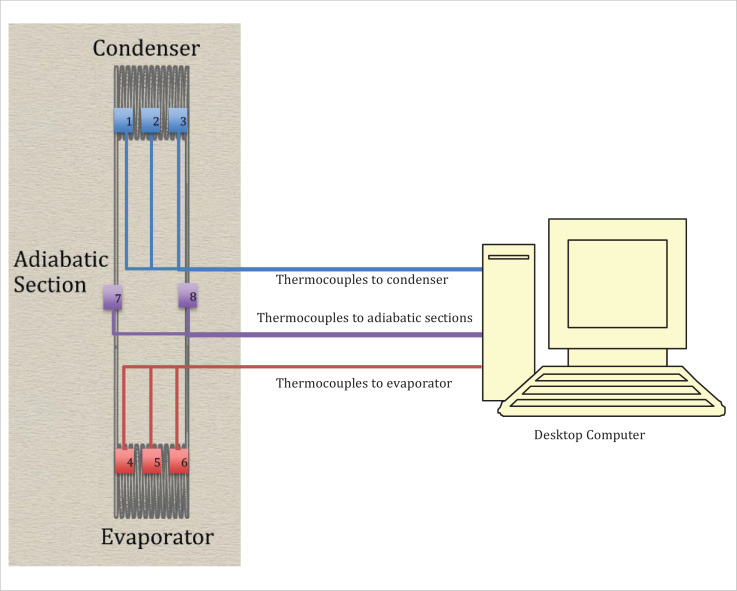
Thermocouple Map for Experimental Set-up.

The Omega K-type thermocouples were connected to the condensers, evaporators, and adiabatic sections as shown in the thermocouple map in Fig. 7. The thermocouples were then connected to the Yokogawa DX 2000 data logger and the desktop computer for the collection of temperature data. The evaporator sections were then subjected to varied heat input with the condensers exposed to the ambient surroundings. Since the original purpose of the helically coiled HCOHPs were to fit around a copper vessel, testing was carried out with hot air blown into the copper vessel and the heat generated via the walls transferred to the evaporators. For this approach three test runs were carried out namely Run 1, 2 and 3. The dimensions of the HCOHP are presented in Table 4 while that of the cylindrical copper vessel can be found in Table 5.

**Table 4:** HCOHP Dimensions.

Parameter	Area (m^2^)	Length (m)
Evaporator	0.02	0.19
Condenser	0.02	0.19
Adiabatic Section	-	0.2

**Table 5:** Dimensions of the Heat Transfer Vessel.

Component	Length (cm)	Inner Diameter (cm)	Outer Diameter (cm)
Copper Vessel	30.00	7.80	8.00

### 2.5 Temperature and power input measurements

**Fig. 8:**
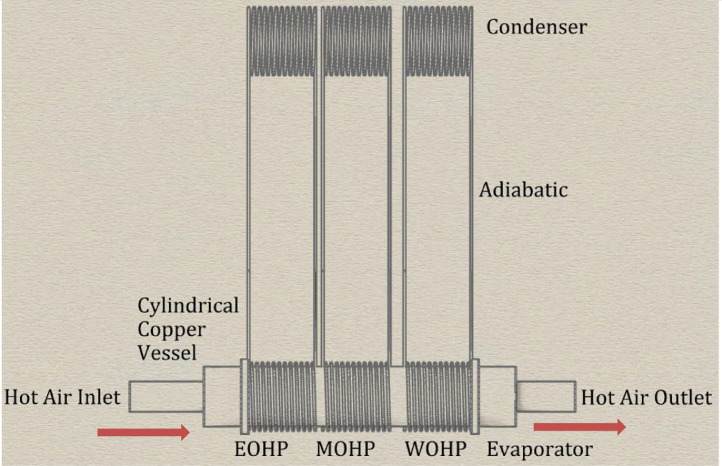
HCOHP testing schematic.

The HCOHPs were oriented vertically with the evaporators at the bottom and the condensers at the top as shown in testing schematic in Fig. 8. The power input to the HCOHPs was achieved using a cylindrical copper vessel integrated with the helically coiled evaporator sections of the HCOHPs as shown in Fig. 7. To ensure that the conditions were the same for all the HCOHPs during testing, the cylindrical copper vessel was pushed through the helical evaporator coils of the three HCOHPs and insulated together using a 20mm thick nitrile rubber thermal insulation material for each test run. OMEGA K-type thermocouples attached to the inner and outer wall surfaces of the cylindrical copper vessel provided the temperature readings of the inner and outer surfaces for the input flux and power to be calculated. To ensure the accuracy of the temperature measurements obtained from the Omega K type thermocouples, they were doubled for each probe point to avert failure. Also the inlet air temperature of the copper vessel was verified with the Omega K type thermocouples using a Sentry ST732 Hotwire Anemometer and AZ8829 data logger to ensure that the measured values were consistent. A final verification of the temperature measurements of the condensers exposed to the ambient surroundings was carried out using the FLIR T640 Infrared camera. The absolute uncertainty of the temperature measurement and verification instruments can be found in Table 6.

**Table 6:** Absolute Uncertainties for the Temperature Measurement Instruments.

Parameter	Measurement Device	Absolute Uncertainty	Units
Temperature	FLIR T640 Infrared Camera	±2	°C
Temperature	Omega K Type Thermocouples	±0.1	°C
Temperature	AZ 8829 sensor and data logger	±0.6 (from -20∼50 °C), ±1.2 (others)	°C
Temperature	Sentry ST 732 Hotwire Anemometer	±2 (from -20∼100 °C)	°C

### 2.6 Relevant design and analysis equations

The rate of heat transfer though the walls of the empty cylindrical copper vessel integrated with the HCOHPs was determined using equation (1) [6].

(1) $$q_{w}=-kA_{s}\frac{dT}{dr}=2\pi \text{Lk}\frac{T_{i}-T_{o}}{\ln\left(r_{o}\;/\;r_{i}\right)}$$

The evaporator heat flux was determined from Fourier's Law given by equation (2) [7].

(2) $$Q=-kA\frac{dT}{dx}$$

Each HCOHP thermal performance was evaluated by determining the thermal resistance (R) using equation (3) obtained from Hao et al. [8].

(3) $$R=\frac{{\bar{T}}_{e}-{\bar{T}}_{c}}{Q}$$

The thermal resistance between the two contacted solid surfaces of the evaporator coils and the cylindrical copper vessel resulting from the surface irregularities and asperities was calculated using equations (4 and 5) obtained from Zhang et al. [9].

(4) $$R_{c}=\frac{T_{v}-T_{evap}}{q_{av}}$$

(5) $$q_{av}=\frac{q_{v}+q_{evap}}{2}$$

Where

• A = cross sectional area, (m^2^)
• $A_{s}$=surface area of cylindrical copper vessel (m^2^)
• k = material thermal conductivity (W/m•K)
• L = length of the material (m)
• Q - heating power input (W)
• $q_{av}$ =the average heat flux of the vessel and evaporator coils (W/m^2^)
• $q_{v}$ = heat flux from the vessel (W/m^2^)
• $q_{evap}$ = heat flux at the evaporator (W/m^2^)
• R = thermal resistance (°C/W)
• $r_{i}$=inner radius of packed bed vessel (m)
• $r_{o}$=outer radius of packed bed vessel (m)
• ${\bar{T}}_{c}$ - Condenser temperature (°C)
• ${\bar{T}}_{e}$ - Evaporator temperature(°C)

## Ethics Statement

Not Applicable

## Declaration of Competing Interest

The authors declare that they have no known competing financial interests or personal relationships which have, or could be perceived to have, influenced the work reported in this article.
